# Distinct bacterial profiles according to structural lung disease in patients undergoing bronchial artery embolization for hemoptysis

**DOI:** 10.3389/fmed.2026.1852948

**Published:** 2026-05-29

**Authors:** Ui Won Ko, Mihyeon Heo, Suyoung Park, Jung Han Hwang, Beomsu Shin

**Affiliations:** 1 Department of Allergy, Pulmonology and Critical Care Medicine, Gil Medical Center, Gachon University College of Medicine, Incheon, Republic of Korea; 2 Department of Radiology, Gil Medical Center, Gachon University College of Medicine, Incheon, Republic of Korea

**Keywords:** bronchiectasis, embolization, hemoptysis, microbiology, polymerase chain reaction, *Pseudomonas aeruginosa*

## Abstract

**Background:**

Hemoptysis frequently prompts emergency bronchial artery embolization (BAE), and bacterial infection is a common and modifiable trigger. As structural lung disease shapes airway microbiology, pathogen profiles may differ according to the structural lung disease phenotype and could inform empiric antibiotic selection in patients undergoing BAE.

**Methods:**

We retrospectively reviewed consecutive patients who underwent BAE for hemoptysis at a tertiary referral center between January 2021 and December 2025. Structural lung disease was stratified into three categories based on computed tomography findings: (1) absence of structural lung disease, (2) bronchiectasis, or (3) emphysema/interstitial lung disease (ILD). Respiratory specimens (sputum or bronchial washing fluid) were evaluated using standard cultures and multiplex polymerase chain reaction (mPCR), when ordered clinically.

**Results:**

A total of 188 patients were included [absence of structural lung disease (*n* = 70), bronchiectasis (*n* = 96), or emphysema/ILD (*n* = 22)]. Overall, 77 patients (41.0%) showed ositive culture or mPCR results. The most commonly identified bacteria were *Streptococcus pneumoniae* (14.3%) in patients without structural lung disease, *Pseudomonas aeruginosa* (29.2%) in patients with bronchiectasis, and *Klebsiella pneumoniae subsp. pneumoniae* (18.2%) in patients with emphysema/ILD. *P. aeruginosa* was more frequent in patients with bronchiectasis than in those without structural lung disease (29.2% vs. 1.4%, *P* < 0.001). *K. pneumoniae* was detected more frequently in 18.2% of patients with emphysema/ILD and in 5.7% of those without structural lung disease (*P* = 0.090).

**Conclusion:**

Patients who underwent BAE showed distinct bacterial profiles according to their underlying structural lung disease. These differences may suggest that tailoring empiric antibiotics according to the structural lung disease phenotype could be considered when an infection is suspected in patients with hemoptysis.

## Introduction

Hemoptysis is a potentially life-threatening respiratory emergency, ranging from mild blood-streaked sputum to massive airway bleeding, often requiring urgent diagnostic evaluation and prompt intervention. In the management of clinically significant or recurrent hemoptysis, bronchial artery embolization (BAE) has become a cornerstone therapy because it is a highly effective and minimally invasive procedure for achieving immediate hemostasis, with reported immediate clinical success rates ranging from 77 to 100% (1–3). However, recurrence remains substantial, with reported rates ranging from approximately 11% at 6 months to 35% at 4–5 years, underscoring the importance of identifying and addressing modifiable precipitating factors (2).

Although anatomical vascular abnormalities are an immediate source of bleeding, bacterial infection is among the most common and potentially modifiable precipitating factors. Infection-associated airway inflammation may exacerbate mucosal injury, promote angiogenesis, and increase vascular friability, thereby triggering or worsening hemoptysis (4). Accordingly, empirical antimicrobial therapy is frequently initiated upon hospital admission when an infectious trigger is suspected. Appropriate empirical antibiotic selection requires a thorough understanding of a patient’s respiratory microbiome. Structural lung diseases shape the distinctive respiratory microbiome and alter local host defenses. Structural lung diseases, such as bronchiectasis, emphysema, and interstitial lung disease (ILD), cause abnormalities in the normal anatomical organization of the respiratory tract, which may increase patient vulnerability to specific and distinctive patterns of bacterial establishment and growth (5–7). In particular, *Pseudomonas aeruginosa* colonization, reported in approximately one-quarter to one-third of adult patients with bronchiectasis, is a recognized marker of severe disease and is associated with accelerated lung function decline, frequent exacerbations, increased hospitalization, and worse clinical outcomes (8–10). Similarly, advanced parenchymal destruction in emphysema and ILD may be associated with altered airway microbiology and increased susceptibility to infection (5, 6). Despite the established association between structural lung disease and airway microbial colonization in stable outpatient populations, microbiological data focusing specifically on patients requiring BAE for hemoptysis remain scarce. This gap poses a practical challenge during emergency care, as inadequate empirical coverage of virulent pathogens may allow persistent infection-driven inflammation, whereas the indiscriminate use of broad-spectrum antibiotics may undermine antimicrobial stewardship and promote multidrug-resistant (MDR) organisms (11, 12). In addition, previous studies have rarely stratified acute hemoptysis microbiology according to rigorous radiological structural lung disease phenotyping, leaving limited evidence to guide phenotype-informed empirical antibiotic selection in this high-risk population.

This study aimed to analyze the respiratory pathogen profiles in patients undergoing BAE for hemoptysis according to the radiologic structural lung disease phenotype. We further sought to determine whether phenotype-specific microbiological patterns could inform empirical antibiotic selection when an infection is suspected and support antimicrobial stewardship.

## Materials and methods

### Study population

**FIGURE 1:**
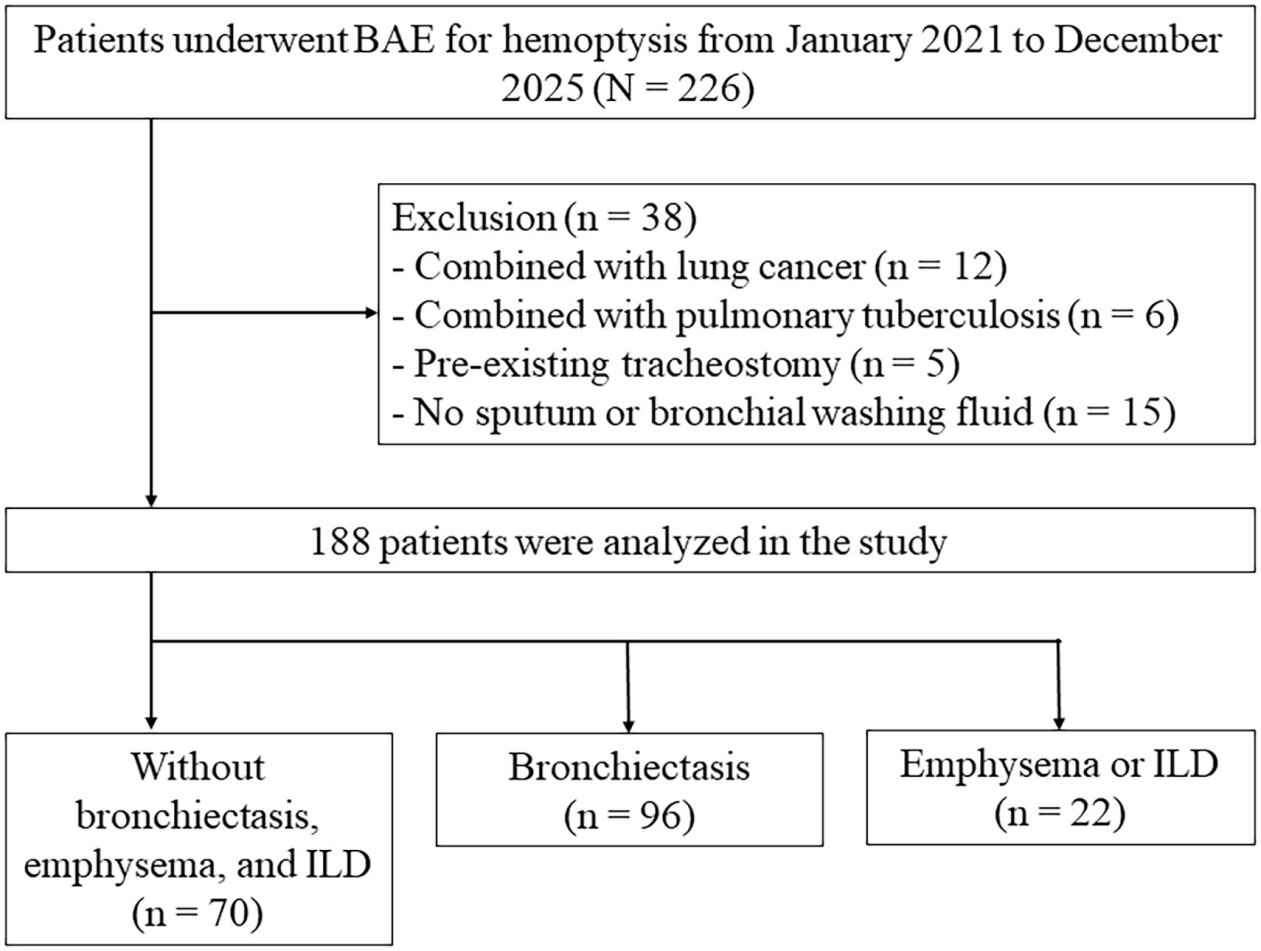
Patient flow chart. BAE, bronchial artery embolization; ILD, interstitial lung disease.

This retrospective observational study included consecutive patients (aged ≥ 18 years) who underwent BAE for the management of hemoptysis at Gil Medical Center (a 1,268-bed tertiary referral hospital in Incheon, South Korea) between January 2021 and December 2025. During the study period, 226 patients underwent BAE for hemoptysis. Patients were excluded if they met any of the following criteria: concomitant lung cancer (*n* = 12), active pulmonary tuberculosis (*n* = 6), pre-existing tracheostomy (*n* = 5), or lack of available respiratory specimens (sputum or bronchial washing fluid) for microbiological evaluation (*n* = 15) (Figure 1). Consequently, 188 patients were included in this study.

### Classification of structural lung disease

Based on the computed tomography (CT) results, patients were categorized according to their underlying structural lung disease. Patients in this study were categorized into three mutually exclusive groups: (1) patients without structural lung disease such as bronchiectasis, emphysema, or ILD (*n* = 70); (2) patients with bronchiectasis (*n* = 96); and (3) patients with emphysema or ILD (*n* = 22) (Figure 1). The presence of structural lung diseases was determined by CT based on standardized criteria. Bronchiectasis was defined according to accepted CT criteria, including bronchial dilatation relative to the accompanying pulmonary artery, lack of normal bronchial tapering, and/or visualization of the bronchi within 1 cm of the pleural surface (13). Emphysema was defined as visual CT evidence of low-attenuation parenchymal destruction using standardized radiologic descriptors (14). ILD was defined as interstitial or fibrotic abnormalities identified on chest CT and documented in clinical radiology reports, including reticulation, ground-glass opacities with interstitial features, traction bronchiectasis, architectural distortion, or honeycombing, in accordance with the contemporary consensus terminology for ILD (15). When bronchiectasis coexisted with emphysema and/or ILD, patients were assigned to the bronchiectasis group, as this phenotype was considered most directly relevant to chronic airway bacterial persistence. In the absence of bronchiectasis, patients with emphysema and/or ILD were assigned to the emphysema/ILD group, whereas those without any of these findings were classified as having no structural lung disease. Emphysema and ILD were combined into a single structural phenotype category for the following reasons: (1) both conditions are characterized by extensive parenchymal destruction, and (2) this combined category represents parenchymal lung disease, in contrast to the airway-centric pathology of bronchiectasis. A phenotype-based classification was used in this study rather than an etiology-based classification because etiologic conditions such as sequelae of tuberculosis, nontuberculous mycobacterial pulmonary disease (NTM-PD), and chronic pulmonary aspergillosis (CPA) frequently overlapped with CT-defined structural abnormalities within the same patient. This classification aligns with the recognized association between the respiratory microbiome and various structural lung diseases (16, 17). Therefore, we selected bronchiectasis, emphysema, and ILD as predefined structural phenotypes based on chest CT because they represent clinically relevant and reproducible abnormalities in patients with hemoptysis and are considered likely to influence airway microbiology and empiric antibiotic decision-making.

### Data collection

Clinical data were obtained through a retrospective review of institutional electronic health records. The collected baseline variables included age, sex, body mass index (BMI), smoking history, and relevant comorbidities such as a previous history of tuberculosis, NTM-PD, CPA, and diabetes mellitus. Serum C-reactive protein (CRP) levels, length of hospital stay, and in-hospital mortality due to hemoptysis were also documented.

### Microbiologic evaluation

Respiratory specimens, specifically sputum and/or bronchial washing fluid, obtained on hospital admission or immediately upon clinical stabilization prior to the interventional procedure, were used for microbiological evaluation. Regarding specimen types, the majority of samples were spontaneously expectorated sputum (*n* = 150/188); bronchial washing fluid (*n* = 38/188) was also obtained from patients who underwent flexible bronchoscopy to localize the bleeding site prior to BAE. All specimens were evaluated for bacterial pathogens via Gram staining and inoculated on BAP, MacConkey, and chocolate agar. Definitive bacterial identification was then conducted using automated VITEK^®^ 2 (bioMérieux Inc., Hazelwood, MO, United States) systems. The samples were classified as Murray–Washington classification degree IV or V (degree IV, 10–25 epithelial cells and > 25 leukocytes; degree V, < 10 epithelial cells and > 25 leukocytes per low-magnification field [ × 100]). Bacterial pathogens were also detected using the Allplex™ PneumoBacter Assay (Seegene Inc., Seoul, South Korea), a multiplex polymerase chain reaction (mPCR) system designed for the simultaneous identification of seven targets: *Chlamydophila pneumoniae, Mycoplasma pneumoniae, Legionella pneumophila, Bordetella pertussis, Bordetella parapertussis, Streptococcus pneumoniae*, and *Haemophilus influenzae*. The mPCR testing was performed exclusively on sputum samples; it was ordered at the discretion of the treating clinicians in routine clinical practice rather than mandated by the study protocol. In addition, because the assay targeted only a predefined panel of seven bacterial pathogens, organisms outside this panel could not be detected by mPCR.

Microbiological positivity was defined as the detection of at least one bacterial pathogen by either conventional culture or mPCR. However, microbiological positivity was not considered synonymous with clinically confirmed active infection, as detected organisms may also represent colonization or persistent airway carriage, particularly in patients with chronic structural lung disease. As multiple organisms could be concurrently identified in a patient, organism-specific frequencies were analyzed on a duplicated-case basis where applicable.

### Embolization procedure and outcomes

BAE was indicated for (1) massive hemoptysis (≥100–200 mL/24 h), (2) clinically significant or recurrent hemoptysis that was refractory to conservative management, or (3) hemoptysis causing hemodynamic instability or respiratory compromise.

Preprocedural contrast-enhanced CT was performed to identify hypertrophied systemic arteries, including those associated with systemic-to-pulmonary shunts. All embolization procedures were performed in the angiography suite by one of the three experienced interventional radiologists. After local anesthesia at the access site, the right or left common femoral artery was punctured under ultrasound guidance, and a 5–6 Fr sheath was inserted. Target arteries were superselected using a 5 Fr diagnostic catheter and a 1.7–2.0 Fr microcatheter (Progreat Lambda, Progreat Alpha; Terumo Interventional Systems, Somerset, NJ, United States). Embolization was performed using particles of greater than 200 μm (Bearing, Embosphere; Merit Medical Systems Inc., South Jordan, UT, United States) and a mixture of *n*-butyl 2-cyanoacrylate (Histoacryl; B. Braun, Melsungen, Germany) and ethiodized oil (Lipiodol Ultra Fluid; Guerbet, Villepinte, France). Hemostasis was achieved at the access site using either manual compression or a vascular closure device. Technical success was defined as the successful selection and embolization of all target arteries with no residual hypertrophied systemic arteries and/or abnormal lung parenchymal staining on completion aortography, whereas failure was indicated by continued or recurrent hemoptysis within 24 h after the first BAE. Recurrence was defined as expectoration of fresh blood during the clinical follow-up period and the requirement for immediate additional treatment such as repeated BAE or surgical treatment (18).

### Statistical analysis

Continuous variables are reported as median values with interquartile ranges (IQR), whereas categorical variables are described as counts (numbers) and percentages (%). The Mann-Whitney U test was used to assess continuous variables, whereas categorical variables were examined using either the chi-square test or Fisher’s exact test, depending on appropriateness. All statistical analyses employed two-sided testing, and significance was set at < 0.05. All statistical analyses were performed using SPSS for Windows (version 27.0; IBM Corp., Armonk, NY).

### Ethics statement

The Institutional Review Board (IRB) of Gil Medical Center, Gachon University College of Medicine, reviewed and approved the study protocol (IRB number: GDIRB 2026-083). The IRB formally exempted this study from the requirement to obtain written informed consent from participants, citing two key factors: the retrospective nature of the research design and the fact that only de-identified clinical data were analyzed.

## Results

### Baseline characteristics of patients with BAE

A total of 188 patients were included in this study; their baseline characteristics are presented in Table 1. The median age was 67 years (IQR, 59–75), 89 patients (47.3%) were male, the median BMI was 22.1 kg/m^2^ (IQR, 19.3–24.7), and 74 patients (39.4%) were ever-smokers. Previous tuberculosis was present in 79 patients (42.0%), NTM-PD in 28 patients (14.9%), CPA in 22 patients (11.7%), and diabetes mellitus in 36 patients (19.1%). The median CRP was 0.51 mg/dL (IQR, 0.09–1.86), the median length of hospital stay was 5 days (IQR, 3–8), and in-hospital mortality occurred in five patients (2.7%).

**TABLE 1:** Baseline characteristics of patients who underwent BAE.

Characteristics	*N* = 188
Age, years	67 (59–75)
Sex, male	89 (47.3)
Body mass index, kg/m^2^	22.1 (19.3–24.7)
Ever-smoker	74 (39.4)
Comorbidities*	
Previous history of tuberculosis	79 (42.0)
Bronchiectasis	96 (51.1)
Emphysema	19 (10.1)
Interstitial lung disease	3 (1.6)
Nontuberculous mycobacterial pulmonary disease	28 (14.9)
Chronic pulmonary aspergillosis	22 (11.7)

Diabetes mellitus	36 (19.1)
C-reactive protein, mg/dL	0.51 (0.09–1.86)
Length of hospital stay, day	5 (3–8)
In-hospital mortality	5 (2.7)

*Cases are duplicated. Values are presented as medians (interquartile ranges) or numbers (%). BAE, bronchial artery embolization.

Compared to patients without structural lung disease, those with bronchiectasis were older (67 years vs. 63 years, P = 0.046), less frequently male (29.2% vs. 58.6%, P < 0.001), had a lower BMI (21.9 kg/m^2^ vs. 23.4 kg/m^2^, P = 0.021), and were less likely to be ever-smokers (19.8% vs. 48.6%, *P* < 0.001). They also had a higher prevalence of previous tuberculosis (49.0% vs. 30.0%, *P* = 0.017) and NTM-PD (24.0% vs. 5.7%, P = 0.001), and had higher CRP levels (0.81 mg/dL vs. 0.16 mg/dL, *P* = 0.003) (Table 2-1).

**TABLE 2-1:** Baseline characteristics of patients who underwent BAE according to the structural lung disease.

Characteristics	Patients without bronchiectasis, emphysema, or ILD (*N* = 70)	Patients with bronchiectasis (*N* = 96)	*P*-value
Age, years	63 (52–76)	67 (61–74)	**0.046**
Sex, male	41 (58.6)	28 (29.2)	**<0.001**
Body mass index, kg/m^2^	23.4 (20.5–26.1)	21.9 (19.5–24.2)	**0.021**
Ever-smoker	34 (48.6)	19 (19.8)	**<0.001**
Comorbidities*			
Previous history of tuberculosis	21 (30.0)	47 (49.0)	**0.017**
Nontuberculous mycobacterial pulmonary disease	4 (5.7)	23 (24.0)	**0.001**
Chronic pulmonary aspergillosis	7 (10.0)	8 (8.3)	0.787
Diabetes mellitus	19 (27.1)	14 (14.6)	0.051

C-reactive protein, mg/dL	0.16 (0.05–1.19)	0.81 (0.18–1.86)	**0.003**
Length of hospital stay, day	5 (3–8)	5 (4–8)	0.321
In-hospital mortality	2 (2.9)	2 (2.1)	>0.999

*Cases are duplicated. Values are presented as medians (interquartile ranges) or numbers (%). The values presented in bold indicate statistical significance (*P* < 0.05). BAE, bronchial artery embolization; ILD, interstitial lung disease.

Patients with emphysema or ILD were older than those without structural lung disease (73 years vs. 63 years, *P* = 0.002), were predominantly male (90.9% vs. 58.6%, *P* = 0.005), had a lower BMI (18.6 kg/m^2^ vs. 23.4 kg/m^2^, *P* < 0.001), and were more likely to be ever-smokers (95.5% vs. 48.6%, P < 0.001). CPA was also more frequent in the emphysema/ILD group (31.8% vs. 10.0%, *P* = 0.035) (Table 2-2).

**TABLE 2-2:** Baseline characteristics of patients who underwent BAE according to the structural lung disease.

Characteristics	Patients without bronchiectasis, emphysema, or ILD (*N* = 70)	Patients with emphysema or ILD (*N* = 22)	*P*-value
Age, years	63 (52–76)	73 (66–82)	**0.002**
Sex, male	41 (58.6)	20 (90.9)	**0.005**
Body mass index, kg/m^2^	23.4 (20.5–26.1)	18.6 (17.3–22.0)	**<0.001**
Ever-smoker	34 (48.6)	21 (95.5)	**<0.001**
Comorbidities*			
Previous history of tuberculosis	21 (30.0)	11 (50.0)	0.123
Nontuberculous mycobacterial pulmonary disease	4 (5.7)	1 (4.5)	>0.999
Chronic pulmonary aspergillosis	7 (10.0)	7 (31.8)	**0.035**
Diabetes mellitus	19 (27.1)	3 (13.6)	0.258

C-reactive protein, mg/dL	0.16 (0.05–1.19)	0.51 (0.12–4.31)	0.139
Length of hospital stay, day	5 (3–8)	6 (3–16)	0.231
In-hospital mortality	2 (2.9)	1 (4.5)	0.564

*Cases are duplicated. Values are presented as medians (interquartile ranges) or numbers (%). The values presented in bold indicate statistical significance (*P* < 0.05). BAE, bronchial artery embolization; ILD, interstitial lung disease.

### Clinical outcomes of BAE

The overall clinical outcomes of the patients who underwent BAE are shown in Table 3. Technical success was achieved with the first BAE in 181 (96.3%) patients. Clinical failure of the first BAE was observed in only seven patients (3.7%). Post-BAE complications were observed in three patients (1.6%). During the median follow-up period of 14.7 (2.6–31.6) months, recurrence of hemoptysis was observed in a total of 19 (10.1%) patients at a median of 1.5 (0.2–12.8) months after the first BAE. Compared to those without structural lung disease, no significant differences in the rates of technical success or clinical failure of the first BAE were observed (Table 3).

**TABLE 3:** Clinical outcomes of BAE.

Outcomes		Bronchiectasis, emphysema, or ILD		
	(*N* = 188)	No (*N* = 70)	Yes (*N* = 118)	*P*-value
Outcomes of the first BAE				
Technical success	181 (96.3)	68 (97.1)	113 (95.8)	>0.999
Failure	7 (3.7)	2 (2.9)	5 (4.2)	>0.999
Complications of the first BAE				
Embolic events	3 (1.6)	1 (1.4)	2 (1.7)	>0.999
Recurrence of hemoptysis	19 (10.1)	7 (10.0)	12 (10.2)	>0.999
Time to recurrence, months				
<1 month (early onset)	8 (4.3)	5 (7.1)	3 (2.5)	0.151
1 month to 1 year	7 (3.7)	2 (2.9)	5 (4.2)	>0.999
>1 year	4 (2.1)	0	4 (3.4)	0.299

Values are presented as numbers (%). BAE, bronchial artery embolization; ILD, interstitial lung disease.

### Microbiologic findings

Overall, 77 of 188 patients (41.0%) had at least one positive result on conventional bacterial culture and/or mPCR. A single bacterium was identified in 65 patients (34.6%), and multiple bacteria were detected in 12 patients (6.4%). The most commonly identified organisms in the study population were *P. aeruginosa* (30/188, 16.0%), *S. pneumoniae* (20/188, 10.6%), *H. influenzae* (18/188, 9.6%), and *Klebsiella pneumoniae* subsp. *pneumoniae* (14/188, 7.4%) (Table 4).

**TABLE 4:** Microbiological characteristics according to the structural lung disease.

Characteristics		Bronchiectasis, emphysema, or ILD		
	(*N* = 188)	No (*N* = 70)	Yes (*N* = 118)	*P*-value
Culture or mPCR positive	77 (41.0)	20 (28.6)	57 (48.3)	**0.009**
Single bacterium	65 (34.6)	14 (20.0)	51 (43.2)	**0.001**
Multiple bacteria	12 (6.4)	6 (8.6)	6 (5.1)	0.368
Detected bacterial species*				
*Pseudomonas aeruginosa*	30 (16.0)	1 (1.4)	29 (24.6)	**<0.001**
*Klebsiella pneumoniae* ssp. *pneumoniae*	14 (7.4)	4 (5.7)	10 (8.5)	0.576
Enterobacter species				
*Enterobacter cloacae*	1 (0.5)	0	1 (0.8)	>0.999
*Enterobacter aerogenes*	1 (0.5)	0	1 (0.8)	>0.999

*Haemophilus influenzae*	18 (9.6)	9 (12.9)	9 (7.6)	0.306
*Streptococcus pneumoniae*	20 (10.6)	10 (14.3)	10 (8.5)	0.229
*Serratia marcescens*	1 (0.5)	1 (1.4)	0	0.372
*Stenotrophomonas maltophilia*	1 (0.5)	1 (1.4)	0	0.372
*Acinetobacter baumannii*	1 (0.5)	0	1 (0.8)	>0.999
*Achromobacter xylosoxidans/denitrificans*	1 (0.5)	0	1 (0.8)	>0.999
*Staphylococcus aureus*	1 (0.5)	0	1 (0.8)	>0.999

*Cases are duplicated. Values are presented as numbers (%). The values presented in bold indicate statistical significance (*P* < 0.05). ILD, interstitial lung disease; mPCR, multiplex polymerase chain reaction.

### Microbiologic findings according to structural lung disease

The most commonly identified bacteria were *S. pneumoniae* (14.3%) in patients without structural lung disease, *P. aeruginosa* (29.2%) in patients with bronchiectasis, and *K. pneumoniae* (18.2%) in patients with emphysema/ILD. The bronchiectasis group demonstrated significantly higher rates of microbiological positivity than the group without structural lung disease (49/96 [51.0%] vs. 20/70 [28.6%], P = 0.004). This difference was mainly driven by the higher rate of single-bacterial detection in the bronchiectasis group (44/96 [45.8%] vs. 14/70 [20.0%], P < 0.001). *P. aeruginosa* was more frequently identified in patients with bronchiectasis than in those without structural lung disease (29.2% vs. 1.4%, P < 0.001). By contrast, the prevalence of other common pathogens including *S. pneumoniae* (9.4% vs. 14.3%, *P* = 0.336), *H. influenzae* (8.3% vs. 12.9%, *P* = 0.438), and *K. pneumoniae* (6.3% vs. 5.7%, P > 0.999), did not differ significantly between the two groups (Table 5-1).

**TABLE 5-1:** Microbiological characteristics of patients who underwent BAE according to the structural lung disease.

Characteristics	Patients without bronchiectasis, emphysema, or ILD (*N* = 70)	Patients with bronchiectasis (*N* = 96)	*P*-value
Culture or mPCR positive	20 (28.6)	49 (51.0)	**0.004**
Single bacterium	14 (20.0)	44 (45.8)	**<0.001**
Multiple bacteria	6 (8.6)	5 (5.2)	0.530
Detected bacterial species*			
*Pseudomonas aeruginosa*	1 (1.4)	28 (29.2)	<0.001
*Klebsiella pneumoniae* ssp. *pneumoniae*	4 (5.7)	6 (6.3)	>0.999
Enterobacter species			
*Enterobacter cloacae*	0	0	NA
*Enterobacter aerogenes*	0	1 (1.0)	>0.999

*Haemophilus influenzae*	9 (12.9)	8 (8.3)	0.438
*Streptococcus pneumoniae*	10 (14.3)	9 (9.4)	0.336
*Serratia marcescens*	1 (1.4)	0	0.422
*Stenotrophomonas maltophilia*	1 (1.4)	0	0.422
*Acinetobacter baumannii*	0	0	NA
*Achromobacter xylosoxidans/denitrificans*	0	1 (1.0)	>0.999
*Staphylococcus aureus*	0	1 (1.0)	>0.999

*Cases are duplicated. Values are presented as numbers (%). The values presented in bold indicate statistical significance (*P* < 0.05). BAE, bronchial artery embolization; ILD, interstitial lung disease; mPCR, multiplex polymerase chain reaction; NA, not applicable.

Although the emphysema/ILD group showed a higher overall microbiological positivity rate, this difference was not statistically significant (36.4% vs. 28.6%, *P* = 0.596). *K. pneumoniae* was detected more frequently in patients with emphysema or ILD than in those without structural lung disease (18.2% vs. 5.7%, *P* = 0.090) (Table 5-2). The proportion of patients who underwent mPCR did not differ significantly between those without structural lung disease and those with bronchiectasis (34/70 [48.6%] vs. 38/96 [39.6%], *P* = 0.270). Similarly, the rate of mPCR was comparable between patients without structural lung disease and those with emphysema or ILD (34/70 [48.6%] vs. 12/22 [54.5%], *P* = 0.807).

**TABLE 5-2:** Microbiological characteristics of patients who underwent BAE according to the structural lung disease.

Characteristics	Patients without bronchiectasis, emphysema, or ILD (*N* = 70)	Patients with emphysema or ILD (*N* = 22)	*P*-value
Culture or mPCR positive	20 (28.6)	8 (36.4)	0.596
Single bacterium	14 (20.0)	7 (31.8)	0.258
Multiple bacteria	6 (8.6)	1 (4.5)	>0.999
Detected bacterial species*			
*Pseudomonas aeruginosa*	1 (1.4)	1 (4.5)	0.423
*Klebsiella pneumoniae* ssp. *pneumoniae*	4 (5.7)	4 (18.2)	0.090
Enterobacter species			
*Enterobacter cloacae*	0	1 (4.5)	0.239
*Enterobacter aerogenes*	0	0	NA

*Haemophilus influenzae*	9 (12.9)	1 (4.5)	0.442
*Streptococcus pneumoniae*	10 (14.3)	1 (4.5)	0.287
*Serratia marcescens*	1 (1.4)	0	>0.999
*Stenotrophomonas maltophilia*	1 (1.4)	0	>0.999
*Acinetobacter baumannii*	0	1 (4.5)	0.239
*Achromobacter xylosoxidans/denitrificans*	0	0	NA
Staphylococcus aureus	0	0	NA

*Cases are duplicated. Values are presented as numbers (%). BAE, bronchial artery embolization; ILD, interstitial lung disease; mPCR, multiplex polymerase chain reaction; NA, not applicable.

## Discussion

In this retrospective study of patients undergoing BAE for hemoptysis, we demonstrated that respiratory bacterial profiles differ substantially according to the underlying structural lung disease. Specifically, *P. aeruginosa* was the predominant pathogen in patients with bronchiectasis, whereas those without structural lung disease more commonly harbored typical community-acquired pathogens such as *S. pneumoniae* and *H. influenzae*. These findings appear biologically plausible and may contribute to the existing literature on chronic airway bacterial colonization or infection.

Bronchiectasis is characterized by impaired mucociliary clearance, chronic neutrophilic airway inflammation, and irreversible dilatation of the bronchial wall. This architectural distortion creates an isolated ecological niche that is highly favorable for opportunistic Gram-negative organisms. The remarkably high prevalence of *P. aeruginosa* (29.2%) among patients with bronchiectasis in this study is consistent with the established pathophysiological knowledge, identifying it as a major pathogen associated with accelerated lung function decline, severe disease phenotypes, and increased exacerbation frequency (19–21). Importantly, previous studies have demonstrated that *P. aeruginosa* colonization is a potent and independent predictor of recurrent hemoptysis after BAE (22), with MDR strains conferring a particularly high risk of clinical treatment failure and subsequent post-BAE hemoptysis recurrence. Therefore, early identification of these resistant pathogens is crucial for implementing targeted eradication strategies to optimize long-term clinical outcomes (23). By contrast, *K. pneumoniae* was detected more frequently in patients with emphysema or ILD than in those without structural lung disease (18.2% vs. 5.7%, *P* = 0.090); however, this finding was not statistically significant and remains exploratory in nature due to the limited subgroup size. Although this observation requires validation in larger cohorts, it may be broadly consistent with some of the emerging evidence regarding the pathophysiological mechanisms of advanced parenchymal diseases. In emphysema and ILD, extensive structural airway disruption, such as bullae formation and honeycombing, severely impairs mucociliary clearance. This architectural distortion causes significantly lower airway dysbiosis, providing an optimal environment for bacterial colonization and a progressive shift in the respiratory microbiome (6, 24). Furthermore, the natural history of the disease in these patients is characterized by inevitable and frequent healthcare utilization secondary to repeated episodes of acute exacerbation. Repeated exposure to systemic corticosteroids and broad-spectrum antibiotics profoundly alters the local microbiome, selectively suppresses normal respiratory flora, and facilitates the survival and overgrowth of opportunistic Gram-negative bacteria, such as *K. pneumoniae* (25, 26). Patients’ demographic characteristics also contributed significantly. Patients with emphysema and ILD were predominantly older and more frequently exhibited frailty and multiple comorbidities. Advanced age is frequently accompanied by immunosenescence and subclinical oropharyngeal dysphagia, which substantially increases the risk of silent microaspiration of the oropharyngeal and gastrointestinal flora into the lower respiratory tract. Once introduced into structurally compromised and immunologically vulnerable lung parenchyma, *K. pneumoniae* can easily establish chronic colonization. By contrast, the predominance of *S. pneumoniae* and *H. influenzae* in patients without structural lung disease is consistent with well-established community-acquired pneumonia microbiology (27–29). This distinct microbiological pattern suggests that hemoptysis in this specific subgroup is more commonly driven by acute endobronchial and parenchymal inflammation, mucosal hyperemia, capillary injury, and tissue necrosis (30, 31). The relatively intact baseline mucociliary escalator in these patients likely prevented the establishment of more virulent opportunistic Gram-negative pathogens (7).

Our findings have potential clinical implications, particularly for antimicrobial stewardship in patients undergoing BAE for hemoptysis. As recurrent airway inflammation and bacterial infection can acutely precipitate or exacerbate hemoptysis by driving local neovascularity, angiogenic growth factor upregulation, and subsequent bronchial artery hypertrophy (4, 32), empirical antibiotics are frequently initiated during hospital admission for BAE. Our data suggest that radiologic structural lung disease phenotyping could help refine and personalize empirical antimicrobial selection. For patients presenting with bronchiectasis, early anti-pseudomonal coverage could be considered given the high microbiological positivity rate and severe risk of hemoptysis recurrence (22, 23). Subsequent de-escalation may warrant consideration once standard culture or mPCR results are available, depending on the patient’s clinical trajectory (33–35). Conversely, standard empiric community-acquired regimens targeting Gram-positive and typical respiratory pathogens may suffice for patients without structural lung abnormalities in many instances. If validated prospectively, this tailored phenotype-informed approach could maximize therapeutic efficacy while directly supporting vital antimicrobial stewardship initiatives by minimizing unnecessary exposure to broad-spectrum agents.

The current study included the use of a well-defined BAE and a systematic, objective comparison of microbiological data stratified by distinct radiologic structural lung disease phenotypes identified via CT imaging. However, this study has some limitations that warrant careful consideration. First, the retrospective, single-center design and specific inclusion of only patients requiring BAE inherently introduced a selection bias, potentially limiting the generalizability of our findings to milder cases of hemoptysis. Accordingly, the microbiological patterns observed in this study should not be assumed to be directly generalizable to patients with milder, self-limited, or conservatively managed hemoptysis. Second, the retrospective nature of our study precluded the inclusion of pre-admission antibiotic history, detailed antimicrobial resistance profiles, systematic viral or fungal assessments, and long-term outcomes. In particular, as prior antimicrobial exposure may have reduced microbiological yield or altered the relative frequency of detected organisms, these results should be interpreted with caution and validated through future prospective research. Third, microbiological positivity in this study should not be interpreted as equivalent to clinically confirmed active infection, because respiratory organism detection in bronchiectasis and other chronic structural lung diseases may reflect chronic colonization or persistent airway carriage rather than the proximate cause of bleeding. However, chronic airway colonization with organisms such as *P. aeruginosa* in bronchiectasis is a well-recognized phenomenon that may persist independently of acute infectious exacerbations (22, 23). Fourth, while bronchial washing samples exhibited a high culture positivity rate, the routine use of bronchoscopy was often restricted in patients with hemoptysis due to underlying comorbidities and hemodynamic instability (Supplementary Table 1). Additionally, mPCR testing was not uniformly performed in all patients and was limited to a predefined panel of seven bacterial targets, which may have influenced pathogen detection rates. The reported microbiological profile should be interpreted as conditioned in part by the characteristics of the diagnostic assay rather than as a comprehensive representation of all potential respiratory pathogens. However, no consensus has been established regarding the routine performance of mPCR in patients with hemoptysis in previous studies. Finally, as previously noted, the relatively small emphysema and ILD subgroups not only limited the statistical power required to robustly detect differences in less common pathogens but also rendered multivariable analyses adjusting for age, smoking, and comorbidities infeasible. Future studies with larger cohorts should analyze emphysema and ILD as separate phenotypic categories to determine whether their microbiological profiles are sufficiently distinct to warrant individualized management strategies. Most published BAE studies have classified patients by etiology rather than by predefined CT-based structural phenotypes; accordingly, emphysema or ILD has rarely been considered a separate predefined structural category in this population (2, 36). Among these phenotypes, bronchiectasis has been consistently reported as one of the most common underlying conditions in patients undergoing BAE for hemoptysis (2, 36, 37). While we utilized a prespecified CT-based assignment rule, the possibility of misclassification remains due to overlapping radiological findings in some patients, which may have attenuated the distinctiveness of microbiological detection across the defined phenotypes.

## Conclusion

Among patients undergoing BAE for hemoptysis, respiratory bacterial profiles differed significantly and predictably according to the underlying structural lung disease. Bronchiectasis was specifically associated with *P. aeruginosa*, whereas patients without underlying structural diseases predominantly presented with typical respiratory pathogens. These exploratory findings provide a rationale for further investigation into phenotype-informed empirical antibiotic strategies when infection is suspected of triggering hemoptysis. Future prospective multicenter studies integrating standardized diagnostic methods, such as advanced molecular sequencing and comprehensive antimicrobial resistance data, are required to validate these findings and formally assess their impact on long-term clinical outcomes.

## Data Availability

The raw data and original contributions presented in this study are available within the article/Supplementary material, and further details can be obtained from the corresponding author upon reasonable request.
