# Supplementary material for: Distinct bacterial profiles according to structural lung disease in patients undergoing bronchial artery embolization for hemoptysis
Source: Front Med (Lausanne). 2026 May 29;13:1852948. doi: 10.3389/fmed.2026.1852948 (PMC13260604; doi:10.3389/fmed.2026.1852948)
Supplement: Supplementary file 1 [file Table_1.docx]

**Supplementary table 1** Distribution of sputum and bronchial washing samples across study groups

|  | Patients without bronchiectasis, emphysema, or ILD | | Patients with bronchiectasis | | Patients with emphysema or ILD | |
| --- | --- | --- | --- | --- | --- | --- |
|  | N = 70 | | N = 96 | | N = 22 | |
|  | Sputum | Bronchial washing | Sputum | Bronchial washing | Sputum | Bronchial washing |
|  | (n = 52) | (n = 18) | (n = 78) | (n = 18) | (n = 20) | (n = 2) |
| Culture positive | 4 (7.7) | 3 (16.7) | 27 (34.6) | 9 (50.0) | 6 (30.0) | 0 (0) |

Values are presented as numbers (%).

ILD, interstitial lung disease
